# Hongfu Yin: from defining the Golden Spike to shaping geobiology

**DOI:** 10.1093/nsr/nwaf210

**Published:** 2025-05-23

**Authors:** Weijie Zhao, Shucheng Xie

**Affiliations:** School of Earth Sciences, China University of Geosciences (Wuhan); School of Earth Sciences, China University of Geosciences (Wuhan)

## Abstract

Professor Hongfu Yin, born in 1935, is one of the pioneers of paleontology and geobiology in China. He and his colleagues proposed the conodont fossil to replace the ammonoid fossil as the index for defining the Permian-Triassic Boundary (PTB) in 1986, ultimately setting this ‘Golden Spike’ at China's Meishan section in 2001 after 15 years of effort. Afterwards, he devoted much time to development of new interdisciplinary research on geobiology in China. Professor Yin is also a devoted teacher who established a productive research group and trained a large number of excellent younger geologists. Recently, Prof. Yin sat down for an interview with *National Science Review* (NSR) and generously shared his stories of the Meishan Golden Spike, the field of geobiology and his personal scientific career.

## Teaser

The 90-year-old pioneer of Chinese paleontology and geobiology reflects on his lifelong research journey.

## THE FIRST INTER-ERATHEM GOLDEN SPIKE IN CHINA

***NSR:*** Your most well-known contribution is the redefinition of the Global Stratotype Section and Point (GSSP) of the PTB, which is an important inter-erathem boundary established on the initiative of Chinese scientists. How did you start research on chronostratigraphic boundaries?

***Yin:*** In the late 20th century, the International Commission on Stratigraphy (ICS, affiliated with UNESCO) recognized the need for a globally unified stratigraphic subdivision framework, making one of its key missions the determination of global standards for the boundaries between chronostratigraphic units, that is, the GSSPs, or more popularly known as the Golden Spikes.

A Golden Spike must meet the highest standards of spatiotemporal precision, and rigorous evaluations, including three levels of voting and final ratification, are needed before it is finally approved. Thus, the successful designation of a GSSP reflects the scientific leadership of the country (or region) where it is located.

The 4.6-billion-year history of Earth is divided into 4 eons, 10 eras, and 22 periods. The Golden Spike at the PTB represents not just the division between two periods, Permian and Triassic, but also marks the transition from the Paleozoic Era to the Mesozoic Era (Table 1). This makes it one of the most important of all GSSPs.

In 1982, the International Working Group on the PTB (PTBWG) was established, with 23 members from various countries. As I had been working on the PTB for around 20 years by then, I was invited to join the PTBWG together with four other Chinese scientists.

***NSR:*** You redefined the PTB GSSP from ammonoid fossils to conodonts. What is a conodont?

***Yin:*** Traditionally, the PTB was defined by the first appearance of the ammonoid fossil *Otoceras woodwardi*. However, through extensive investigations on tens of sections in China and analysis of global data, I, together with Kexin Zhang and other colleagues, found that the ammonoid fossil lacked global distribution. In contrast, conodonts demonstrated both worldwide distribution and superior isochronous stratigraphic precision. Thus, during the 1986 Brescia conference, we proposed that the conodont *Hindeodus parvus* should replace *O. woodwardi* as the index fossil to define the PTB.

**Figure 1:**
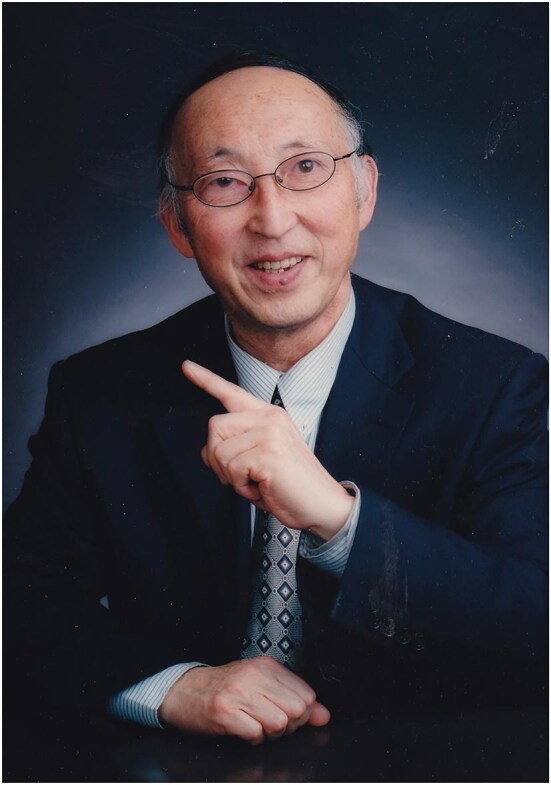
Professor Hongfu Yin (Photo taken in 2006 and provided by Prof. Yin).

Conodonts are generally 0.1–0.5 mm in size and are shaped like fish teeth, which is the source of their name, based on the Greek for ‘cone-tooth’. These microfossils are believed to be skeletal parts of certain extinct marine animals. Unfortunately, the exact taxonomic position of the animal is unclear.

Due to their wide occurrence in Paleozoic and Mesozoic strata and their relatively short stratigraphic ranges, conodonts commonly make excellent index fossils and have been increasingly used as the index fossil for the base of the Triassic Period.

***NSR:*** Conodonts were officially ratified to be the index fossil of the PTB in 2001, 15 years after your initial proposal in 1986. What happened during these years? How did the general attitude of the international geoscience community evolve?

***Yin:*** It was indeed a ‘long trudge’ to make the Meishan section the GSSP of the PTB. Our 1986 proposal received a widespread positive response from the geoscience community, but we faced notable opposition from the then-chairman of the PTBWG. Over the following several years, I observed steadily growing support for our approach, but the PTBWG did not organize any meetings to discuss this issue.

**Table 1.** The setting of the Permian-Triassic boundary (PTB).

Eon	Era	Period	Epoch	Stage
Phanerozoic	Mesozoic	Triassic	Upper	Rhaetian
				Norian
				Carnian
			Middle	Ladinian
				Anisian
			Lower	Olenekian
				Induan
	Paleozoic	Permian	Lopingian	Changhsingian
				Wuchiapingian
			Guadalupian	Capitanian
				Wordian
				Roadian
(To be continued)				

A pivotal moment came at the 1993 Calgary Conference when the chairman of the Triassic Subcommission called for a re-election of the PTBWG. Due to our leadership in advancing the conodont definition, I was elected the new chairman. Then, a preliminary vote was taken in 1995, showing support from a strong majority of PTBWG members for both the Meishan section as the candidate GSSP and conodonts as the index fossil. Building on this momentum, I spearheaded the formal proposal that was published just prior to the 30th Congress of the International Union of Geological Sciences (IUGS) in 1996.

However, the voting process was unexpectedly delayed due to a boycott initiative against the Meishan section. At the time, an international research team comprising geoscientists from China, the United States, Germany, and Russia was conducting studies at the terrestrial PTB Dalongkou section of the Xinjiang Autonomous Region of China. A disagreement among the team members escalated into a formal protest against the Meishan section as the PTB candidate.

Their objection stemmed from access restrictions—similar to Dalongkou, Meishan had not been officially opened to foreign researchers, despite hundreds of international scientists having already visited Meishan without restrictions. The dissenting members sent formal letters of protest to the chairman of the International Commission on Stratigraphy (ICS), all voting members of the PTBWG, and members of both the Permian and Triassic Subcommissions.

As a result of the boycott event, in late 1996, the ICS published updated guidelines for GSSP definition in the journal *Episodes*, requiring GSSP candidate sections to guarantee open access to all stratigraphers worldwide, in order that all researchers would be free to conduct long-term research at the chosen site under the recognition of relevant authorities. The journal *Science* also paid attention to these events and published two relevant news reports in 1996 and 1997.

I still remember our extensive efforts at persuasion, discussion and communication with relevant scientists. Through all these efforts, the above-mentioned organizations ultimately declared their disagreement with the boycott of the Meishan section, and continued to support me as the leader of the PTBWG. Their endorsement came with the explicit condition that the GSSP candidates must be open to all researchers holding a valid visa.

To meet this requirement, we undertook a lot of measures to provide open access to Changxing County, where the Meishan section is located. In September of 1999, the Chinese government announced the opening of Changxing County to all foreigners, and the Meishan section then finally qualified to compete for the Golden Spike of the PTB.

**Figure 2:**
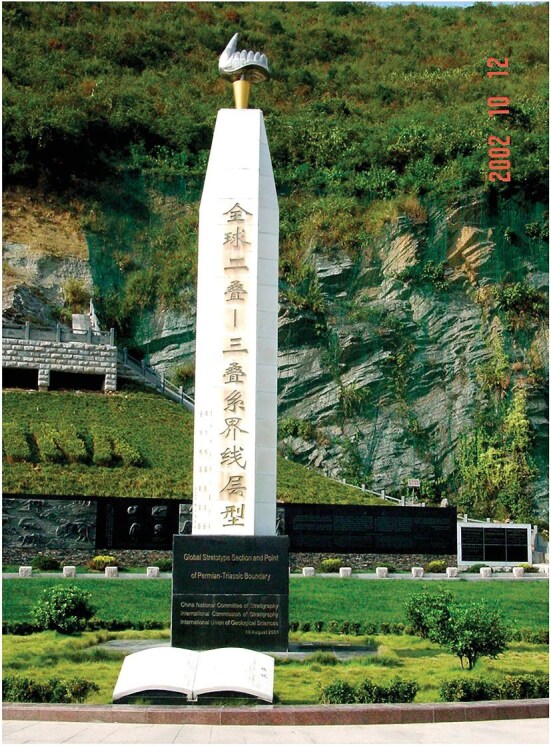
The monument of the GSSP of PTB established at Meishan of Zhejiang, China, with the model of the conodont *Hindeodus parvus* seen on the top (Photo provided by Prof. Yin).

After three rounds of voting and final approval, the Meishan section and the conodont *H. parvus* were confirmed as the GSSP section and the index fossil of the PTB in 2001:

- The 1st vote, by the Working Group on the Permian-Triassic Boundary in January of 2000, resulted in a 20–3 majority.
- The 2nd vote, by the ICS Subcommission on Triassic Stratigraphy in May of 2000, resulted in a 22–2 majority.
- The 3rd vote, by the International Commission on Stratigraphy in November of 2000, yielded a unanimous 17–0.
- Final approval, by the International Union of Geological Sciences, followed in March of 2001.

I was also delighted to see that, in October of 2022, the Meishan section was selected as one of the first 100 global geological heritage sites.

***NSR:*** To date, have all Golden Spikes in geological history been formally ratified? What key challenges remain in this field of research?

***Yin:*** There are a total of 102 Golden Spikes, and 82 of them have been established, with 11 located in China. These geochronological milestones, marked by GSSPs, form the foundation for global stratigraphic subdivision and correlation.

However, with most GSSPs defined, the era of GSSP establishment as the primary focus of the Phanerozoic Eon research has concluded. The focus has pivoted to two major new frontiers: establishing GSSPs for the Cryptozoic Eon, and pursuing higher-precision Phanerozoic stratigraphy. These studies will support the most cutting-edge research directions of geology, including the fields of deep sea, deep Earth, deep space, and Earth system science.

## GEOBIOLOGY: A FAST-EVOLVING FIELD

***NSR:*** In addition to the Golden Spike of the PTB, what are other important scientific achievements you have made?

***Yin:*** After the establishment of the Golden Spike of the PTB at Meishan, I shifted my research from paleontology to biogeology and geobiology. Biogeology is the interdisciplinary field of geology (solid Earth science) and biology, and geobiology aims to integrate geosciences with life sciences. Since the rapid development of geobiology, biogeology has been gradually integrated with it.

I am honored to share that my research group at the China University of Geosciences (Wuhan) won the National Award of Natural Sciences in 2008 for our pioneering contributions to biogeology. This recognition stemmed from a series of books we published in this field. For example, *The Palaeobiogeography of China*, which was published in 1994, may be the first paleobiogeography monograph concerning a broad region of the world. Through detailed regional paleobiogeographic descriptions of all Phanerozoic periods of China, this book established systematic criteria for the paleobiogeography of tropical, temperate and polar zones. The English edition of this book was commented upon by Prof. Anthony Hallam as ‘an invaluable guide to a subject of global significance’.

The core task of geobiology is to explore the interaction and co-evolutionary processes of organisms and the environment.

—Hongfu Yin

In addition to this publication, I also led the investigations on biometallogenesis and ecological stratigraphy, which are both important branches of biogeology.

***NSR:*** Please comment more about geobiology. What are the basic scientific questions of this field?

***Yin:*** As I mentioned earlier, geobiology is an interdisciplinary field between Earth science and life sciences, with biogeology as one of its branches. Earth science concerns exceptionally grand temporal and spatial scopes, and it is quite different from life sciences in terms of research subjects, research methods and research goals. Therefore, the integration of these two fundamental disciplines is very difficult and requires the construction of an integrated framework.

The core task of geobiology is to explore the interaction and co-evolutionary processes of organisms and the environment. This establishes geobiology as an important part of Earth-system science. Since their origin on Earth, organisms have been interacting with and influencing the environment. These interactions have driven organism-environment co-evolution, thus shaping the biosphere into the colorful world we see today.

***NSR:*** How has geobiology research evolved over the past decades, in terms of its research goals and methodologies, and the support it received?

***Yin:*** Geobiology began to emerge in the early 20th century. Its early development was marked by the proposals of several major concepts, including the concepts of the *biosphere* by Vernadsky in 1926, *geobiology* in 1934, and the *Gaia hypothesis* by Lovelock in 1978. In 1987, the International Geosphere-Biosphere Program was started. It pioneered the study of Earth as an integrated system, and it demonstrated the necessity of establishing geobiology as an independent interdisciplinary science.

The new millennium has witnessed rapid development of the field of geobiology, although it is still in its developmental adolescence compared to more established fields such as geochemistry or geophysics. Some significant developments include the launching of professional journals (for example, *Geobiology* in 2003), publication of a series of monographs and textbooks, foundation of the Geobiology Society in 2016, convocation of regular scientific conferences, establishment of departments of geobiology in universities, and the availability of stable long-term support by multiple research and funding institutions.

In China, I noticed that the early researchers in the field of geobiology were basically paleontologists. Paleontology is a traditional discipline focusing on the identification and classification of fossils, with its textbooks largely filled by taxonomical descriptions of macrofossils. However, as a fast-developing interdisciplinary discipline, geobiology has diverged significantly from paleontology through its evolution, exhibiting fundamental differences in research topics and methodologies. In geobiology, the emphasis is more on the microbial world of both the past and the present, and it more actively embraces modern research technologies. For example, gas chromatography-mass spectrometry is widely used to detect and characterize ‘chemical fossils’, such as the lipid biomarkers preserved in sedimentary rocks and other samples, to identify ancient microorganisms.

**Figure 3:**
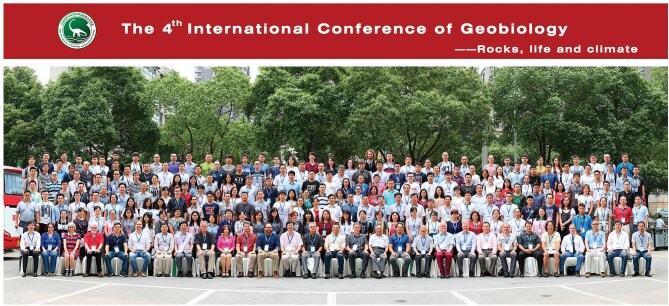
Group photo of the 4th International Conferences of Geobiology held at China University of Geosciences (Wuhan) in 2017 (Photo provided by Prof. Yin).

***NSR:*** What are the most important scientific questions currently captivating your attention in geobiology?

***Yin:*** To me, geobiology is a fascinating and promising field, and I am currently most concerned about three topics.

The first one is how microorganisms have shaped global environmental change in geological history. Microorganisms account for over 90% of all living species. They have dominated Earth for over 3.5 billion years―which is 6 times longer than the history of animal life―and they are described as the kings of the Earth and the engine of life. As dominant organisms, their interactions and reciprocal influences with the environment have greatly shaped our planet through geological time. One example of their utility for global change studies is that, because the composition of microbial membrane lipids sensitively varies in response to temperature and other environmental factors, multiple microbial lipids such as the long-chain alkenones in algae and the tetraether compounds in bacteria and archaea have been employed as temperature proxies. Our current understanding of microorganisms in geological history is still limited, but I have confidence that, with the continuous emergence of novel techniques and new ideas, further rapid developments in this field can be expected.

The second issue in which I am interested is geobiological processes during major geological events such as the Early Proterozoic Great Oxygenation Event and Phanerozoic mass extinctions. For example, the hyperthermal climate conditions at the PTB resulted in widespread anoxia and the mass extinction of macro-organisms, but during the same period, some microbes such as cyanobacteria and green sulfur bacteria significantly expanded. Such geobiological phenomena are the result of intensive interactions among the atmosphere, hydrosphere and biosphere, and represent the best subjects for learning about organism-environment co-evolutionary mechanisms and gaining insights into how to deal with present-day climate change.

The last issue in which I am interested is the nature of the biosphere in extreme environments. Geobiological research is now extending into extreme environments such as deep space, deep sea and deep Earth. Such studies will significantly broaden the temporal and spatial boundaries of the biosphere and biological processes, with wide-ranging applications in resource exploitation and environmental protection. Particularly, these extreme environments host microorganisms equipped with specific enzymes to perform various functions, making them a huge gene pool with potential applications across industrial, agricultural and other socio-economic fields.

***NSR:*** Geobiology focuses primarily on Earth’s history. How can it enlighten humanity's present and future?

***Yin:*** We should learn about the present and future from the past. Today, with humans sitting at the top of the food chain, is the ‘present’ stage of organism-environment co-evolutionary history. I would like to offer some examples to explain how geobiological knowledge might influence the present and future.

First, we can evaluate humans’ environmental impacts from geobiological records. The human-environment interaction is so strong that it may influence the ‘survival or destruction’ of human beings, and geobiological research can help us to quantify this interaction and its impact.

Second, geobiology helps to explore the relationship between climate and biodiversity. Sudden climate change can trigger significant changes in biodiversity or even a mass extinction, and it is very likely that we are on the brink of another biodiversity crisis. The mass extinction events in geological history provide us natural experiments from which to identify the threshold of climate change that can trigger a biodiversity crisis.

Third, geobiological investigations of human-microorganism interactions will contribute to securing global health. Microorganisms, including viruses, are an important part of natural ecosystems that cannot and should not be eliminated by humans. We need to learn to coexist with microbes. Interactions between micro- and macro-organisms over geological time will provide us with valuable knowledge.

***NSR:*** How is geobiology progressing in China? What are your suggestions for the future development of this field?

***Yin:*** Geobiology was introduced into China around the turn of the century and has been rapidly developing since then, basically in sync with the international community. China has long been regarded internationally as ‘one of the global leaders in the development of geobiology’. Our group at China University of Geosciences (Wuhan) as well as groups at China University of Geosciences (Beijing), Northwest University and the Chinese Academy of Sciences (CAS) have delved into geobiological research for many years and made major achievements. The CAS and the National Natural Science Foundation of China (NSFC) also attach great importance to this discipline and to the provision of long-term stable support for it.

I am quite positive about the future development of geobiology in China. My major suggestion is that we should readjust the research framework and its focus by paying more attention to microorganisms in geological history, and by linking research projects more closely with national and societal needs. Particularly, the leading research and funding agencies should organize some big programs in geobiology to facilitate innovative breakthroughs in both theoretical and applied aspects, so that China can maintain global leadership in geobiology and Earth system science.

## RETROSPECTS AND ADVICES

***NSR:*** In your personal academic journey, is there any perception that you think is particularly meaningful and that you especially want to share with the younger generation?

***Yin:*** I graduated from the Beijing Institute of Geology—the predecessor of China University of Geosciences (Beijing) and China University of Geosciences (Wuhan)—in 1961 at the age of 26, and became a teaching assistant at the same university. But what may surprise you is that I remained in this position for the following 17 years, until China's Reform and Opening-up started in 1978 and I was finally promoted to lecturer at age 43.

During this unusually long period, while maintaining a full teaching load, I undertook no research projects and published nothing except for my graduation thesis. These were undoubtedly the leanest years of my academic career. However, I refused to be discouraged but instead prepared about ten scientific papers although there was nowhere to publish them because all concerned publication outlets had been shut down during these years. When academic normalization returned around 1978, all of these papers were quickly published. Thanks to this publication record and my persistence, in 1978, I was among the first batch of scholars selected to study in the United States as a visiting scholar. In 1980, I was promoted to associate professor.

In retrospect, I think the reason I was able to get back to scientific research in my 40s is that I had an unshakable conviction during those days that the difficulties of our nation were temporary. China will ultimately return to prioritizing development, when our knowledge will serve our motherland's construction. This belief has guided my entire scientific research career, and I would like to share this with the younger generations.

***NSR:*** What suggestions do you have for young scholars?

***Yin:*** I would like to share four sentences with them:

Cultivating oneself and serving the country is our belief;

Innovation and seeking truth are the roads of life.

In research one must race against time,

But not be eager for instant benefits and fame.

## Notes

Weijie Zhao is an NSR news and science editor based in Beijing; Shucheng Xie is a Professor at the School of Earth Sciences, China University of Geosciences (Wuhan).

